# Rational design, synthesis, characterization and catalytic properties of high-quality low-silica hierarchical FAU- and LTA-type zeolites

**DOI:** 10.1038/s41598-018-34479-4

**Published:** 2018-11-02

**Authors:** Rajesh K. Parsapur, Parasuraman Selvam

**Affiliations:** 1 National Centre for Catalysis Research and Department of Chemistry, Indian Institute of Technology-Madras, Chennai, 600036 India; 2 School of Chemical Engineering and Analytical Science, The University of Manchester, Manchester, M13 9PL United Kingdom; 3 Department of Chemical and Process Engineering, University of Surrey, Guildford, Surrey, GU2 7XH United Kingdom

## Abstract

Despite the development of several synthetic strategies employing various templates as (pore) structure directing agents, the preparation of high-quality aluminum-rich hierarchical zeolites (designated as ZH) with Si/Al < 5 from its molecular precursors is still a challenge to the scientific community. For the first time, we report here, a successful synthesis methodology for the preparation of hierarchical zeolites, having FAU and LTA topologies with uniform micropores and mesopores by a rationally designed method. Here, a stable supramolecular self-assembly was achieved under the challenging synthesis conditions by tailoring the zeolitization process, viz., by a homogeneous nucleation and a multi-step crystallization. This has resulted in regular mesoporosity in FAU-type zeolites and a unique mesoporosity in LTA-type zeolites, hitherto not reported so far.

## Introduction

The successful development of zeolites and their innovative application in refineries and separation processes has renewed the industrial catalysis to a greater extent^1–5^. In particular, zeolites with FAU (Zeolites X and Y) and LTA (Zeolite-A) topologies have potential applications in various industrial processes^5,6^. Despite such remarkable success, these materials suffer from severe mass-transfer limitations owing to sluggish diffusion characteristics in the narrow pore-channels^7–9^. From the perspective of modern crude oil reserves with high boiling fractions and feedstock from unconventional resources, it is important to develop effective zeolite catalysts for the efficient utilization of existing resources. Therefore, the novel materials that can overcome such constraints can have profound impact on industrial research. In this regard, numerous efforts have been undertaken to induce secondary porosity in to such zeolitic structures by employing various post-synthetic modifications^10–14^. However, such treatments can result in poorly interconnected pores which can led to undesirable reverse hierarchy^15,16^. On the other hand, the multi-step nanocasting procedures used for the synthesis of such materials are expensive^17–19^. In addition, the mesoporosity was also induced by the repetitive branching of FAU/EMT intergrowths^20^. Lately, the synthesis of nanocrystalline zeolites have attracted a great deal of interest owing to their excellent physico-chemical properties^21–24^. However, they too have drawbacks, viz., tedious separation methods, loss of crystallinity, etc.^14^. On the other hand, the concept of supramolecular templating has been adopted for the preparation of hierarchical zeolites^25–27^ wherein the existence of two or more types of pores of different sizes, i.e., micropores and mesopores, can possibly overcome the diffusion problems in small pores. Nevertheless, engineering such pore structures through this approach is tricky owing to challenging synthesis conditions. However, few reported the synthesis of such structures by using surfactants as structure directing agents^28–32^. At this juncture, it is noteworthy that most of these efforts are confined to high silica (Si/Al > 10) materials having MFI and BEA framework structures. On the other hand, attempts to impart similar pore structures in FAU and LTA-type zeolites have met only limited success^33–35^. Indeed, unlike the high silica zeolites, the FAU and LTA-type structures are routinely synthesized in the absence of organic templates, from high-alkaline, dense-gel mixtures with Na_2_O-Al_2_O_3_-SiO_2_-H_2_O quaternary systems. Therefore, the dispersion of supramolecular moieties in such viscous gels is extremely difficult^23,33,36^.

It is, however, well known that the process of zeolitization involves numerous metastable phases which make the synthesis susceptible to various parameters including temperature, precursor concentration, stirring rate, etc.^37^. In particular, the nucleation and crystallization process of FAU and LTA-type framework structures is more complex than the high silica frameworks^38,39^. The aluminosilicate species can spontaneously polymerize while mixing the silica and alumina precursors resulting in the rapid formation of pre-crystalline nuclei with irregular dimensions^39^. Such particles with unequal sizes have dissimilar growth kinetics and can result in quick growth of the large crystals by Ostwald ripening. The typical non-covalent interactions of supramolecular self-assembly cannot withstand such rapid crystal growth and hence the collapse of the mesostructure. Therefore, tailoring the crystallization process in order to achieve the desired pore-architecture in zeolites is still a major challenge. In the present work, we have overcome this incongruity for the synthesis of hierarchical FAU (ZH-X, ZH-Y) and LTA-type (ZH-A) structures by a controlled zeolitization process. In the designed approach, the polymerization kinetics of the aluminosilicates has been decreased in order to regulate the zeolitization and to form a stable crystalline mesophase. The initially organized mesophase is gradually crystallized in the further stages to obtain a one dimensional and a three dimensional hierarchical organizations of FAU and LTA-type zeolites respectively.

## Experimental

### Starting materials

All the chemicals *viz*. colloidal silica (Ludox-AS 40, 40 wt.% SiO_2_, Aldrich), Al metal powder (325 mesh, 99%, Merck), Aluminium hydroxide (98%, Aldrich), dimethyloctadecyl[3-(trimethoxysilyl)propyl]ammonium chloride (DOAC; 60 wt.% in methanol, Acros Organics), NaOH (97%, Merck) are directly used without any modifications.

### Synthesis of ZH-Y

In a typical synthesis, sol-A was prepared by mixing 1.0 *g* of NaOH in 16.0 *g* of H_2_O, followed by the addition of 5.0 *g* of colloidal silica under vigorous stirring. Alternately, sol-B was prepared by dissolving 1.14 *g* of NaOH in 6.0 *g* of H_2_O, followed by the addition of 0.52 *g* of Al(OH)_3_. Both the precursor solutions were stirred for 15 *min* and then the sol-B was added drop wise to the sol-A on the ice bath (0–4 °C) under vigorous stirring (>800 *rpm*). The stirring was continued for 1 *h*, followed by the addition of 1.0 *g* of organosilane (DOAC) under stirring. The mixture with final composition 1Al_2_O_3_: 10SiO_2_: 8.0Na_2_O: 0.36DOAC: 420H_2_O was further stirred for 2 *h* and aged at 35 °C for 24 *h*. The aged gel was crystallized by hydrothermal treatment at 50 °C and 100 °C for 24 *h* each. The resulting solids were washed with distilled water, filtered and dried at 100 °C, followed by the calcination in air at 550 °C for 6 *h* at a heating rate of 1 °C *min*^*–1*^ to obtain highly crystalline ZH-Y.

### Synthesis of ZH-X

ZH-X was synthesized by similar procedure as that of ZH-Y except for the change in the synthesis composition and crystallization temperature. Al metal powder was used as aluminium precursor. The initial gels with synthesis composition 1.0Al_2_O_3_: 9.0SiO_2_: 9.0Na_2_O: 0.36DOAC: 380H_2_O were aged at room temperature for 24 *h* and hydrothermally treated at 50 °C for 24 *h* and 75 °C for 48 *h*. The finally obtained solids were washed with distilled water, filtered and dried at 100 °C, followed by the calcination in air at 550 °C for 6 *h* at a heating rate of 1 °C *min*^*–1*^ to obtain ZH-X.

### Synthesis of ZH-A

Sol A was prepared separately by dissolving 1.0 *g* of NaOH in 11.2 *g* of H_2_O, followed by the addition of 3.0 *g* of colloidal silica. The solution was stirred vigorously for 15 *min*. Meanwhile, sol B was prepared by dissolving 1.72 *g* of NaOH in 11.0 *g* of H_2_O, followed by careful addition of 0.54 *g* of Al metal powder. Both the precursor solutions were stirred for 15 *min* and then mixed to each other on an ice bath under vigorous stirring. The stirring was continued for 1 *h*, followed by the addition of 1.0 *g* of organosilane (DOAC). The mixture with final composition 1 Al_2_O_3_: 2 SiO_2_: 3.4 Na_2_O: 0.12 DOAC: 140 H_2_O was further stirred for 2 *h* and aged at room temperature for 24 *h*. The aged gel was crystallized by hydrothermal treatment at 50 °C for 24 *h* and 75 °C for 12 *h*. The resulting solids were washed with distilled water, filtered and dried at 100 °C, followed by the calcination in air at 550 °C for 6 *h* at a heating rate of 1 °C *min*^*–1*^ to obtain ZH-A. For comparison, conventional zeolites viz., zeolite-Y (Z-Y), zeolite-X (Z-X) and zeolite-A (Z-A) have been prepared by using similar procedures without the addition of organosilane surfactant.

## Characterization

Powder X-ray diffraction (XRD) measurements were carried out on Bruker D8 Advance X-ray diffractometer with Cu *K*_*α*_ (*λ* = 1.5405 *Å*) radiation source operating at 40 *kV* and 30 *mA*. The Le Bail fit and Rietveld refinements of the diffraction patterns were carried out by using TOPAS software. N_2_ physisorption isotherms were obtained at 77 *K* using Micrometrics ASAP 2020 surface area analyzer, in which catalysts were degassed at 573 *K* for 8 *h* prior to measurements. Specific surface areas (*S*_BET_) of the samples were calculated using Brunauer–Emmett–Teller (BET) method, whereas the micropore (*D*_HK_) and mesopore size distribution (*D*_BJH_) curves were obtained from Horvath-Kawazoe (HK) and Barrett-Joyner-Halenda (BJH) methods, respectively. Transmission electron microscope (TEM) images and selected area diffraction (SAED) patterns were obtained with 2100 JEOL microscope operated at 200 *keV*. Temperature programmed desorption of ammonia (NH_3_-TPD) was performed on Micromeritics Autochem-II chemisorption analyser. Samples were activated at 550 °C for 1 *h* in a helium flow, later they were cooled and maintained at 120 °C prior to their exposure to ammonia vapour, followed by purging with helium for 30 *min*. Desorption of ammonia was performed by heating the reactor at a uniform rate of 10 °C *min*^*–*1^.

Pyridine *in situ* Diffuse Reflectance Infrared Fourier Transformation Spectroscopy (DRIFTS) was performed using Bruker Tensor-27 FT-IR instrument in conjunction with Harrick high vacuum cell in praying mantis reaction chamber. Samples were outgassed at 400 °C for 4 *h* and then cooled to 30 °C under dynamic vacuum. The back ground spectrum was measured prior to pyridine sorption followed by the sample spectra at various temperatures. Thermogravimetric analyses and Differential Scanning Calorimetric analyses of the samples were performed at a heating rate of at a heating rate of 20 °C *min*^–1^ on TA instruments QSDT-600 thermogravimetric analyser. The Dynamic Light Scattering studies were performed using Horiba Partica LA 950 instrument (Scattering angle: 173°, output power: 5 *mW* and wavelength: 650 *nm*).

## Catalytic Activity Studies

Vapour phase *tertiary-*butylation of phenol was carried out in a fixed-bed down flow reactor using 0.5 *g* of zeolite sample. The reactor set-up was pre-heated to 350 °C in the flow of air for 2 *h* followed by cooling to desired reaction temperature using N_2_. The ratio of reactants, weight hour space velocity and Si/Al ratio of the catalysts has been varied in order to study the activity of the catalyst. Nitrogen was used as carrier gas and liquid injection (Miclins) pump was used to feed the mixture of reactants. The transformed gaseous products are condensed in an ice bath and the resulted liquids were collected every hour. The products, viz., *ortho*- *tertiary*-butyl phenol (2-*t*-BP), *para*-*tertiary*-butyl phenol (4-*t*-BP) and 2,4-*di-tertiary*-butyl phenol (2,4-*di*-*t*-BP), were analysed using Perkin-Elmer gas chromatograph with a ZB-1 capillary column.

## Results and Discussion

### Formation mechanism

**Figure 1:**
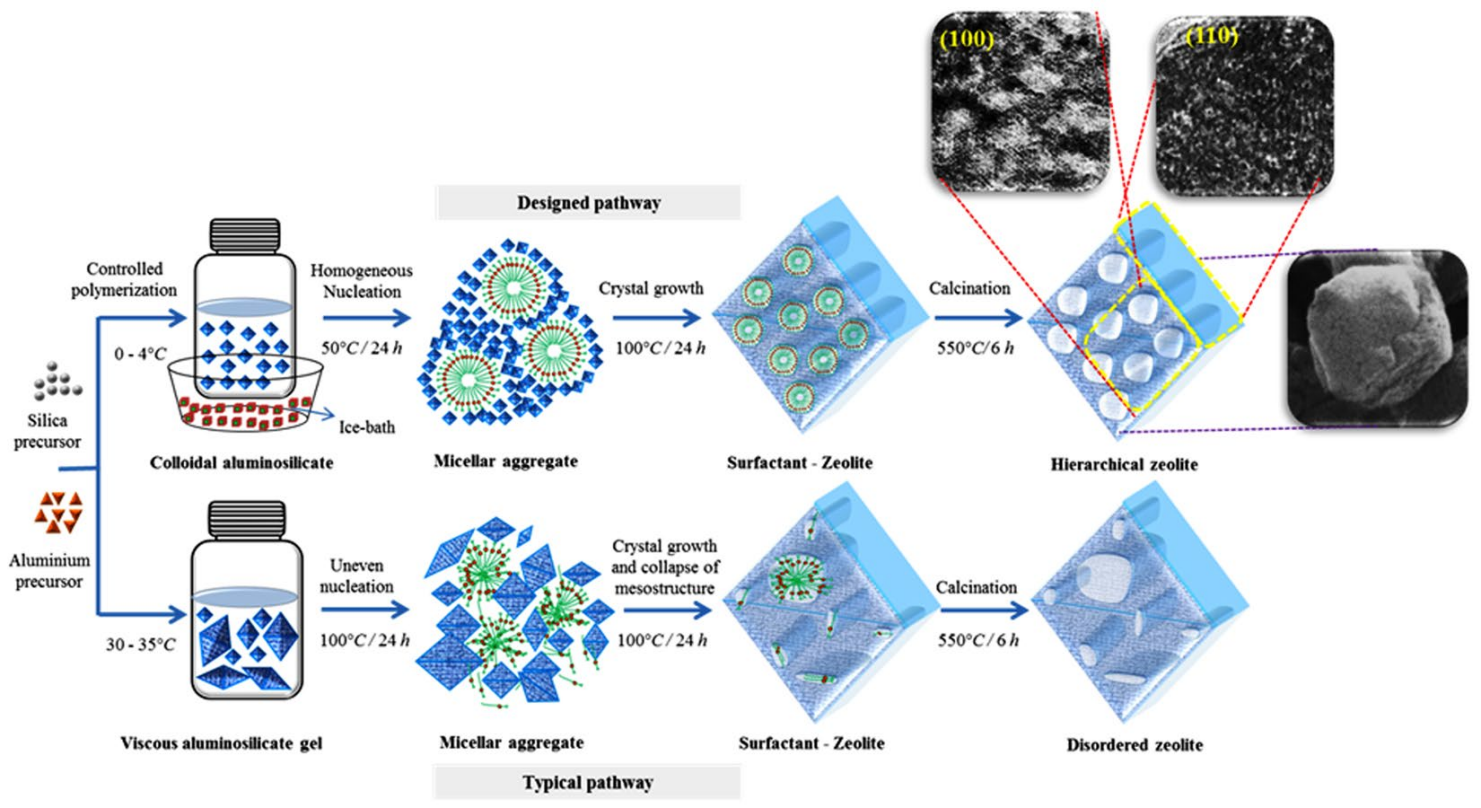
Proposed mechanism for the synthesis of hierarchical FAU-type zeolites.

**Figure 2:**
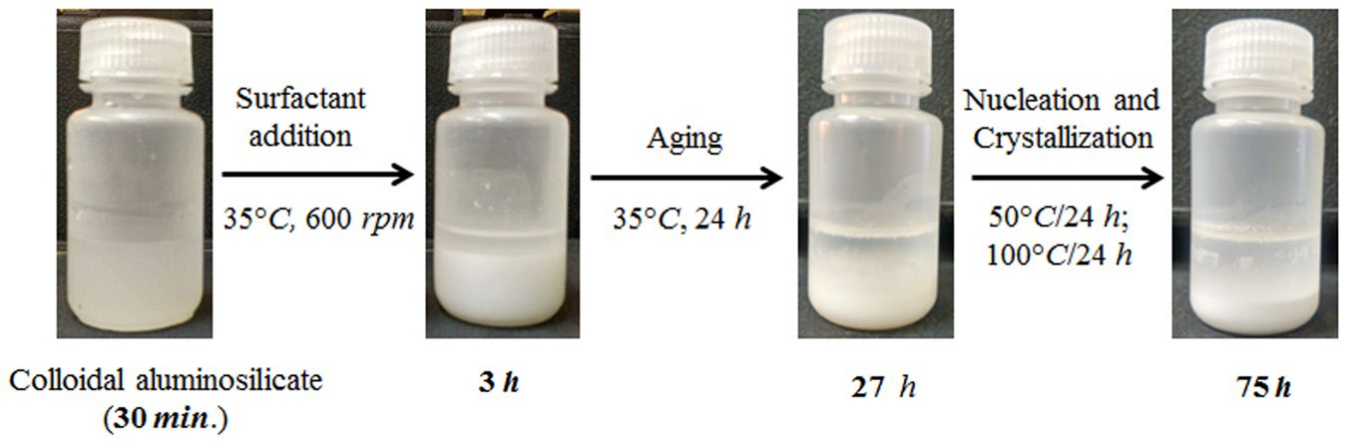
Synthesis mixture (digital photographs) of ZH-Y at various stages of preparation.

In the designed strategy, a large number of homogeneous nuclei have been generated by employing a low-temperature polymerization process^21^. In the initial step, the alkali solutions of silica and alumina were freshly prepared and were mixed on an ice bath (0–4 °C) under vigorous stirring. Such conditions can favor the nucleation over crystal growth as the activation energy needed for the latter is higher^40,41^. In addition, the low temperatures can decrease the kinetics of polymerization resulting in the formation of homogeneous pre-crystalline nuclei^21^. Such population of nuclei with uniform sizes can have similar surface energies and parallel growth kinetics and hence are sterically stabilized. Such phenomenon can ease the uneven crystal growth and assist in the controlled and confined zeolitization around the micellar aggregates without collapsing the organized mesophase (Figs 1 and 2). Furthermore, the cooperative improvement of the mesostructure and framework crystallinity was further supported by the stable Si-C covalent interactions of organosilane. Moreover, the nucleation and crystal growth phenomena of zeolites are highly sensitive to crystallization temperatures^36,39^. Therefore, we have employed a two-step crystallization process for the facile fabrication of hierarchical structure. The synthesis mixture was hydrothermally treated at two different temperatures apart from aging. In the initial step, the nucleation was encouraged over crystal growth by maintaining at low-temperatures (50 °C), which is followed by complete zeolitization at higher temperatures (75–100 °C).

### Characterization results

**Figure 3:**
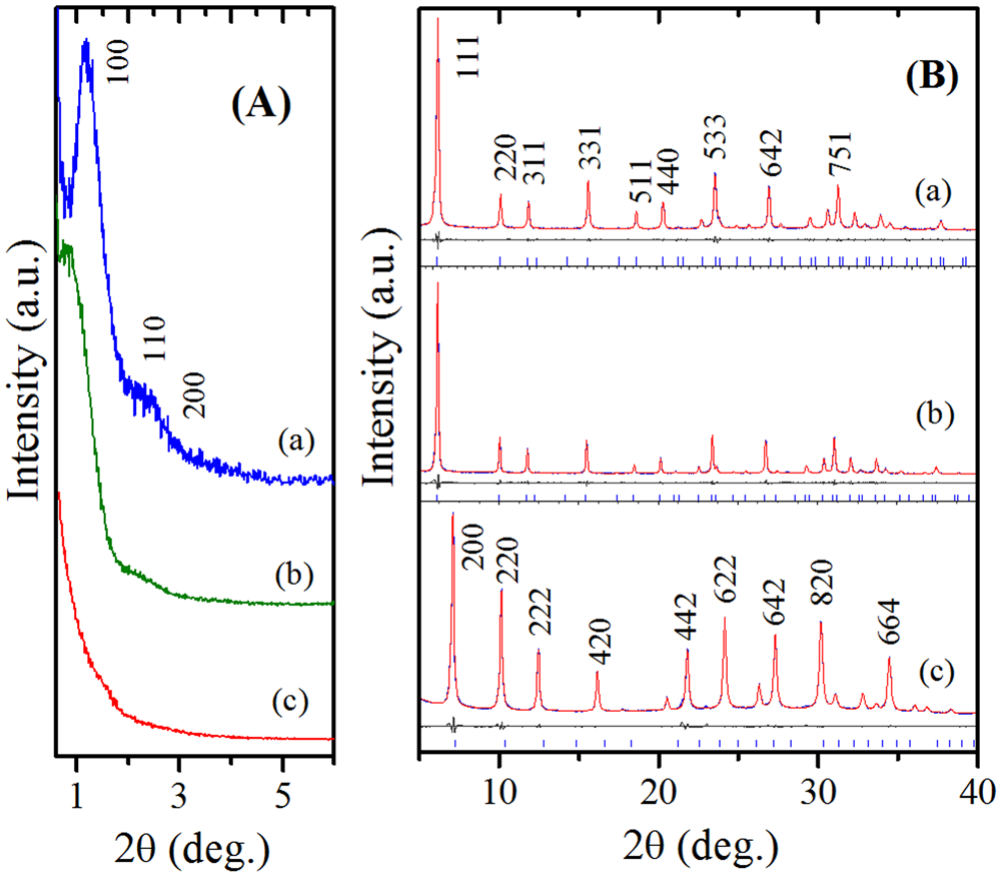
Low-angle (**A**) and high-angle (Rietveld-refined) XRD patterns (**B**) of: (a) ZH-Y; (b) ZH-X; (c) ZH-A.

Figure 3 depicts the XRD patterns of various zeolites. The samples, ZH-Y and ZH-X have shown characteristic Bragg’s reflections in the low-angle region typical of ordered mesoporous materials, whereas the sample, ZH-A has shown broad peak-like feature in the low-angle region indicating disordered mesopore structure. Figure 3B illustrates the Rietveld refined XRD patterns of various zeolites, which show all the reflections characteristic of cubic crystal symmetry with FAU topology (*ICDD card no*. 38–0239, LTA; *ICDD Card No*. 89–3859). The unit cell parameters of ZH-Y (24.64 Å), ZH-X (24.96 Å) and ZH-A (24.61 Å) were obtained by fitting the diffraction patterns with Fd $\bar{3}$ m and Fd $\bar{3}$ c space groups in the 2θ region of 5° to 40° by Le Bail method. The presence of two distinct patterns at low angle and high angle indicate the presence of periodicity at mesoscale (*a*_0_ ~ 10.0 *nm*) as well as atomic level (*a*_0_ ~ 2.5 *nm*). In addition, the diffraction patterns further confirm the absence of impurity phases and/or amorphous phase domains in the samples.

**Figure 4:**
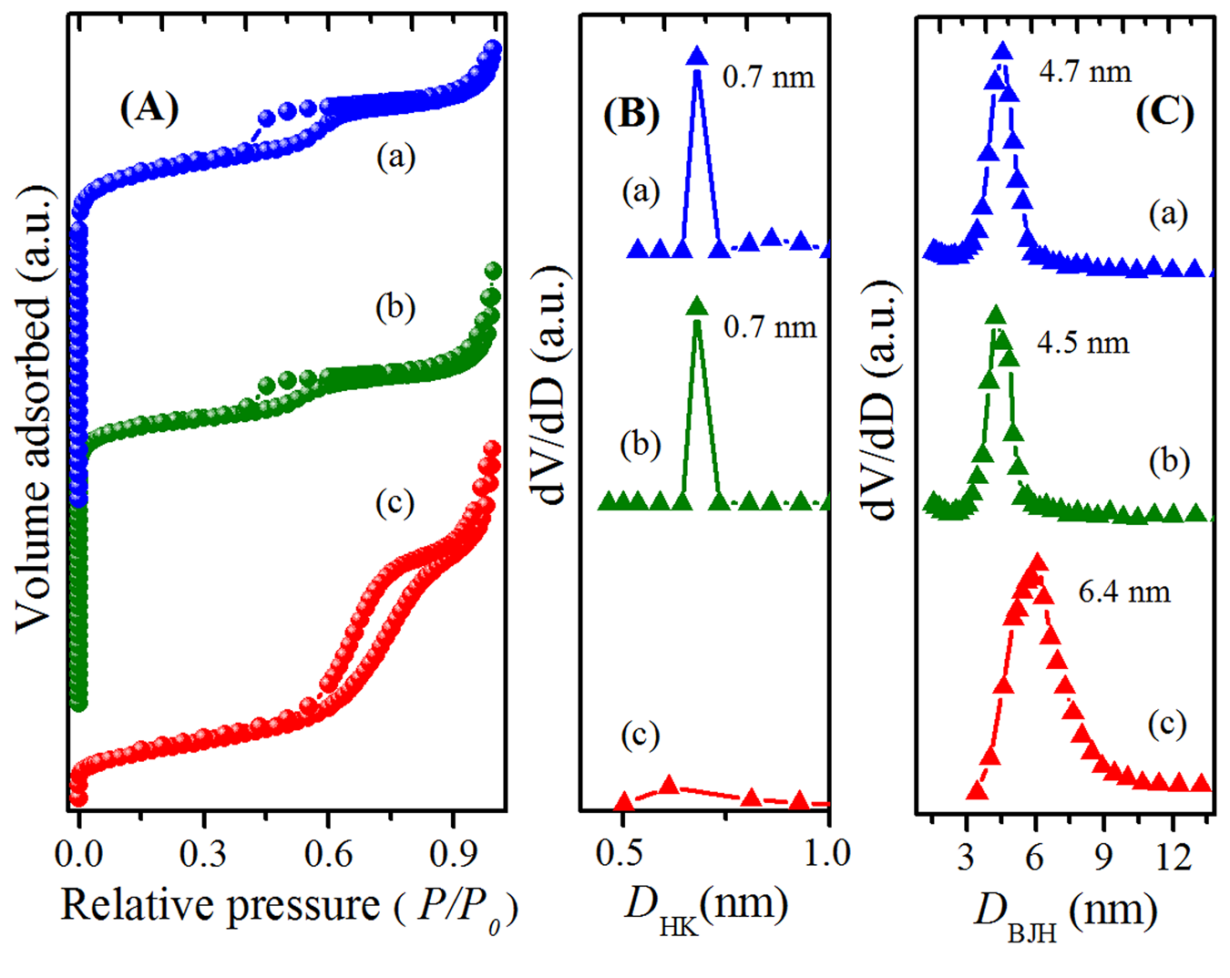
N_2_ sorption isotherms (**A**), micropore (**B**) and mesopore (**C**) size distributions of: (a) ZH-Y; (b) ZH-X; (c) ZH-A.

Figure 4A presents the N_2_ sorption isotherms of the hierarchical zeolites, which show a combination of type-I and type-IV isotherms indicating the presence of micropores in co-existence with the mesopores. The isotherms have shown sharp rise at relative pressure (*P/P*_0_) < 1, which indicate that the zeolitic microporosity is preserved while fabricating the material. This is further supported by the micropore-size distributions (Fig. 4B) obtained by Horvath-Kawazoe (HK) method. In addition, the samples have shown H1 hysteresis corresponding to the condensation phenomenon in the uniform mesopores, which is further evident from the narrow mesopore-size distributions obtained by Barrett-Joyner-Halenda (BJH) method (Fig. 4C). The average pore-size of the zeolite is higher in case of ZH-A owing to the expansion and realignment of micelles under such conditions^32^. However, the H1 hysteresis of ZH-A is well-defined (SBA-like) indicating the cylindrical mesopores formed by the negative imprints of micellar aggregates. The structural and textural properties of the prepared zeolites are shown in Table 1.

**Table 1:** Physicochemical properties of various zeolites.

Sample	Si/Al^a^		*a*_0_ *(nm)*		S_BET_ (*m*^2^ *g*^*−*1^)^d^		*D* (*nm*)^e^		*V*_*p*_ *(cm*^3^ *g*^*−*1^)^f^		*h*_*W*_ *(nm)* ^g^
	XRF	NMR	Crystal^b^	Mesopore^c^	Micro	Total	Micro	Meso	Micro	Total	
ZH-Y	2.1	2.2	2.46	8.90	372	634	0.7	4.7	0.20	0.43	4.20
Z-Y	2.3	2.3	—	—	382	400	0.7	—	0.24	0.25	—
ZH-X	1.4	1.5	2.49	11.60	367	521	0.7	4.5	0.19	0.36	7.10
Z-X	1.1	1.2	—	—	404	412	0.7	—	0.24	0.26	—
ZH-A	1.0	1.0	2.61	—	—	95	40.4	6.4	0.01	0.22	—
Z-A	1.0	1.0	—	—	—	5	—	—	0.004	0.01	—

^a^Si/Al ratio determined from ^29^Si MAS-NMR; ^b^Crystal lattice constant by Rietveld refinement; ^c^Mesopore structure lattice constant calculated using *1/d*^2^ = *4/3 (h*^2^ + *hk* + *k*^2^*/a*^2^); ^d^BET surface area; ^e^Micro and mesopore size by HK and BJH methods; ^f^Pore volume; ^g^Mesopore-wall-thickness, *h*_*w*_ = *a*_0_ – *D*_*BJH*_.

**Figure 5:**
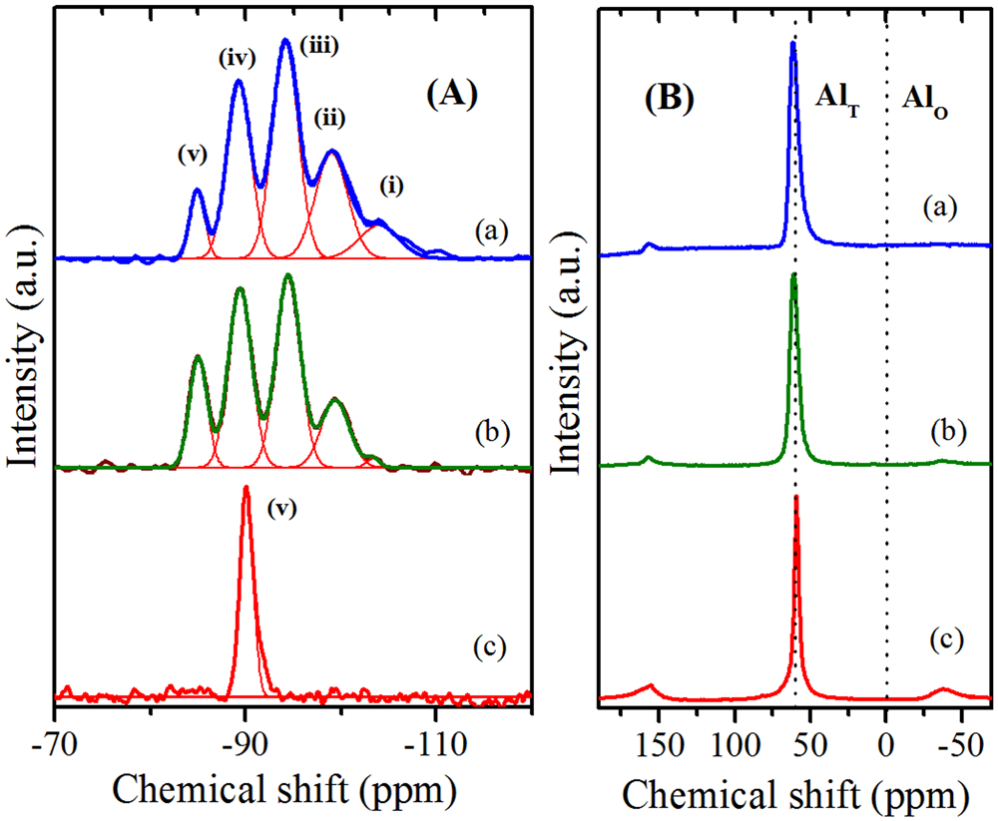
29Si MAS NMR (**A**) and 27Al MAS NMR (**B**) spectra of: (a) ZH-Y; (b) ZH-X; (c) ZH-A.

Figure 5 and Table 2 depicts the MAS-NMR spectra and chemical shift values of various hierarchical zeolites. The deconvolution of the ^29^Si MAS-NMR spectra of ZH-X and ZH-Y zeolites has given characteristic 5 major resonances (i-v) corresponding to the presence of Si atoms in the 4Si(0Al), 3Si(1Al), 2Si(2Al), 1Si(3Al) and 0Si(4Al) environments respectively^42^. The substitution of Al atom in to the silicon coordination sphere changes the chemical shift values by ~5 *ppm*. In contrast, the spectra of ZH-A has shown only one major shift around −90 *ppm* indicating the presence of Si atom in single environment 0Si(4Al), strictly following the Lowenstein’s rule^43^. The Si/Al ratios of the ZH-Y (2.1), ZH-X (1.4) and ZH-A (1.0) determined by ^29^Si MAS-NMR are in good agreement with ICP-OES data. Figure 5B depicts the ^27^Al MAS-NMR spectra of various zeolites which show a single peak around ‘60 *ppm*’ indicating the presence of aluminum exclusively in the tetrahedral coordination (Al_T_) inside the framework. The lack of significant peak at ‘0 *ppm*’ indicates the absence of octahedral aluminum (Al_O_).

**Table 2:** NMR Chemical shift values of various zeolites.

Catalyst	^29^Si MAS-NMR (*ppm*)					^27^Al MAS-NMR (*ppm*)
	4Si(0Al) (i)	3Si(1Al) (ii)	2Si(2Al) (iii)	1Si(3Al) (iv)	0Si(4Al) (v)	0Al(4Si) (Al_T_)
ZH-Y	104.1	99.2	94.2	89.2	84.9	61.2
ZH-X	104.0	99.3	94.4	89.4	85.0	61.0
ZH-A	90.0	—	—	—	—	59.1

**Figure 6:**
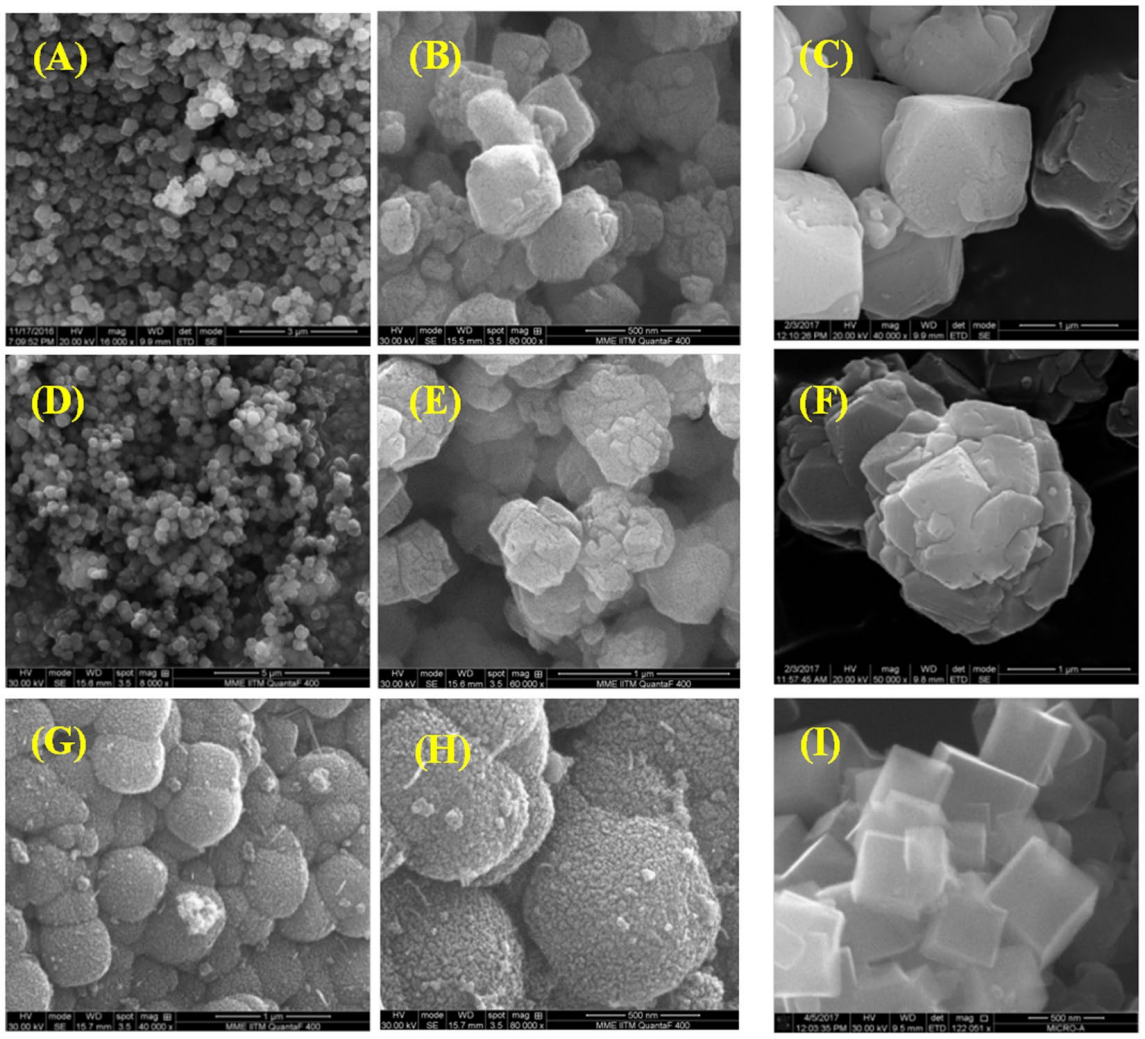
SEM images of various zeolites: ZH-Y (**A,B**) and Z-Y (**C**); ZH-X (**D,E**) and Z-X (**F**); (c) ZH-A (**G,H**) and Z-A (**I**).

**Figure 7:**
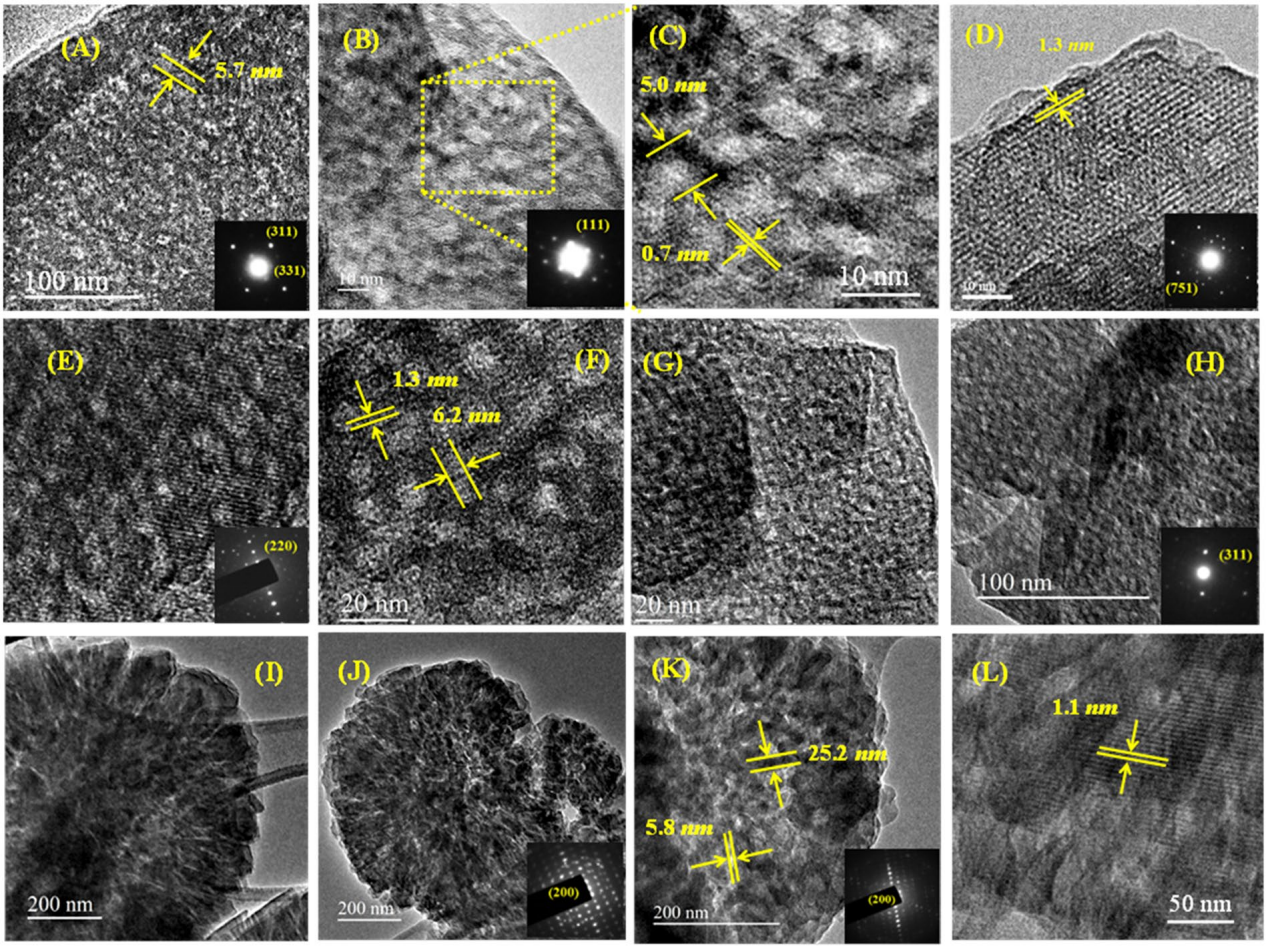
TEM micrographs and SAED patterns of ZH-Y (**A**–**D**), ZH-X (**E**–**H**) and ZH-A (**I**–**L**).

Figure 6 displays SEM images of the hierarchical zeolites and their bulk counterparts. The samples have shown octahedral and cuboid morphologies characteristic of FAU (ZH-X and ZH-Y) and LTA-type (ZH-A) structures respectively. In addition, the high magnification SEM of ZH-A has shown that the crystal is formed by the assembly of smaller nanocrystals. Figure 7 depicts the TEM images of various samples which show three distinct periodic patterns corresponding to the micropores (<1 *nm*), lattice fringes (~1.2 *nm*) and mesopores (~5–10 *nm*). In particular, ZH-Y has clearly shown the presence of regular mesopore channels in the parallel and perpendicular directions of the mesopore axis indicating the high quality of the material. In addition, one can clearly observe the presence of zeolitic frameworks within the mesopore walls of the materials to form a hierarchical organization. Although, the mesopores are not well-ordered as in case of ordered mesoporous materials, however, they are regularly arranged throughout the crystal domain to form short-range order. Furthermore, the crystalline nature of the samples is known from the SAED patterns which reflect the cubic crystal symmetries. This is further supported by the presence of lattice fringes of ~1.3 *nm* and ~1.1 *nm* corresponding to (111) and (200) planes of FAU and LTA topologies respectively.

**Figure 8:**
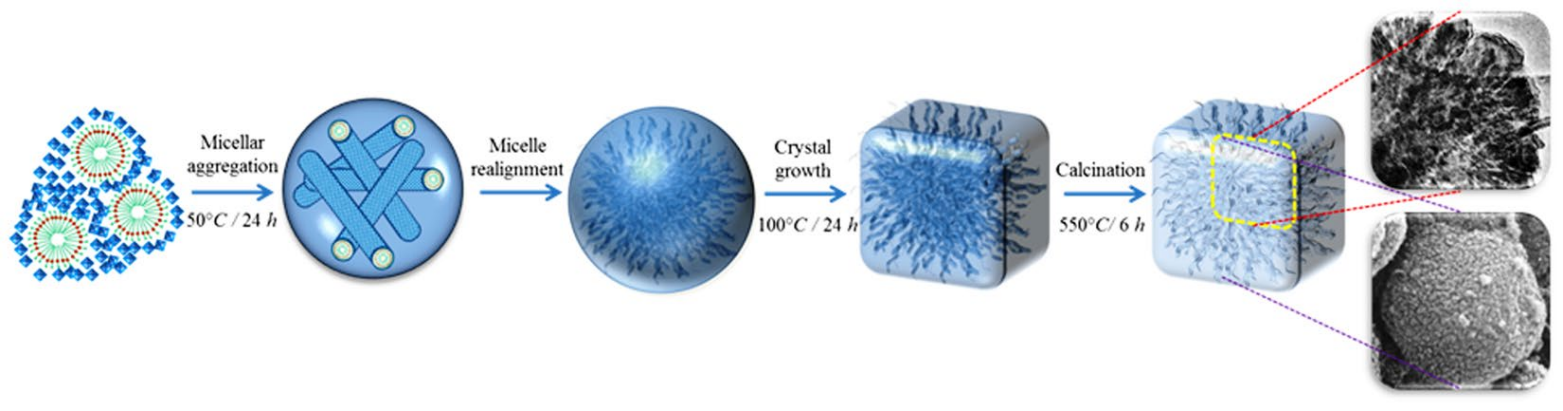
Proposed mechanism for the synthesis of ZH-A with centrosymmetric radial mesopore arrangement.

**Figure 9:**
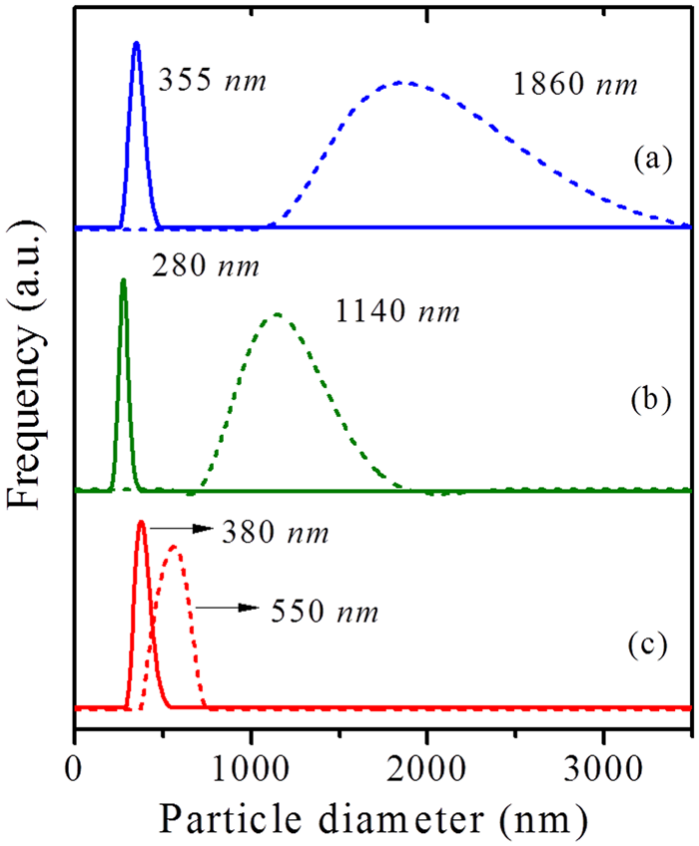
DLS particle size distribution profiles (hierarchical - solid lines; bulk - dotted lines): (**a**) ZH-Y and Z-Y; (**b**) ZH-X and Z-X; (**c**) ZH-A and Z-A.

On the other hand, the TEM of ZH-A (Fig. 7I–L) has shown interesting pattern of centrosymmetric, radial mesopore arrangement as seen in *Radiolarians* (marine organisms). This could be attributed to realignment of randomly formed micellar aggregates in to radial array to minimize the surface free energy in a pre-crystalline spherical particle^44,45^. Such mesostructured patterns are typical in self-assembly process of silica nanoparticles^46^. In a similar way, TEM and SEM images of ZH-A show the formation of mesoporous cuboids (~400 *nm*) by the organization of uniform sized zeolite nanocrystals (20–30 *nm*) around the micellar aggregates (~6 *nm*). This phenomenon clearly supports the proposed mechanism which indicates that the initially formed zeolite nanocrystals can form stable micellar aggregates with surfactants and are then crystallized gradually (Fig. 8). However, unlike the soft organic/inorganic hybrids, the radial mesostructure is not well-defined due to crystal growth of ZH-A. Furthermore, the SEM images of ZH-A (Fig. 6G,H) have clearly revealed the effect of controlled zeolitization as the particle size of samples is much uniform (300–400 *nm*) and lower than the conventional zeolites prepared under similar conditions. This is further confirmed by the dynamic light scattering (DLS) studies which reveal the uniform hydrodynamic diameters of hierarchical zeolites (Fig. 9).

**Figure 10:**
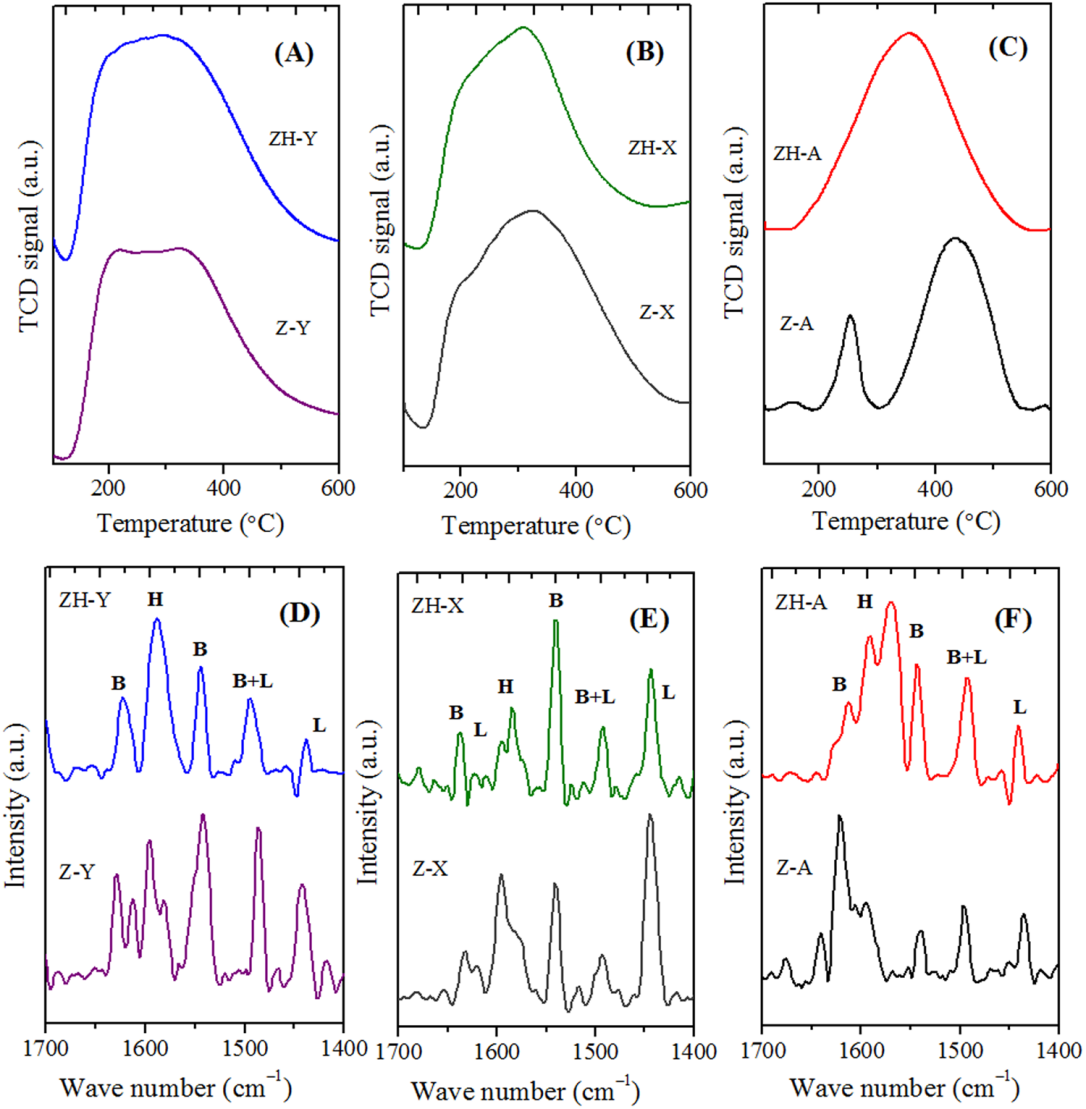
NH_3_-TPD traces (**A**–**C**) and pyridine DRIFT spectra (**D**–**F**) of various zeolites.

**Figure 11:**
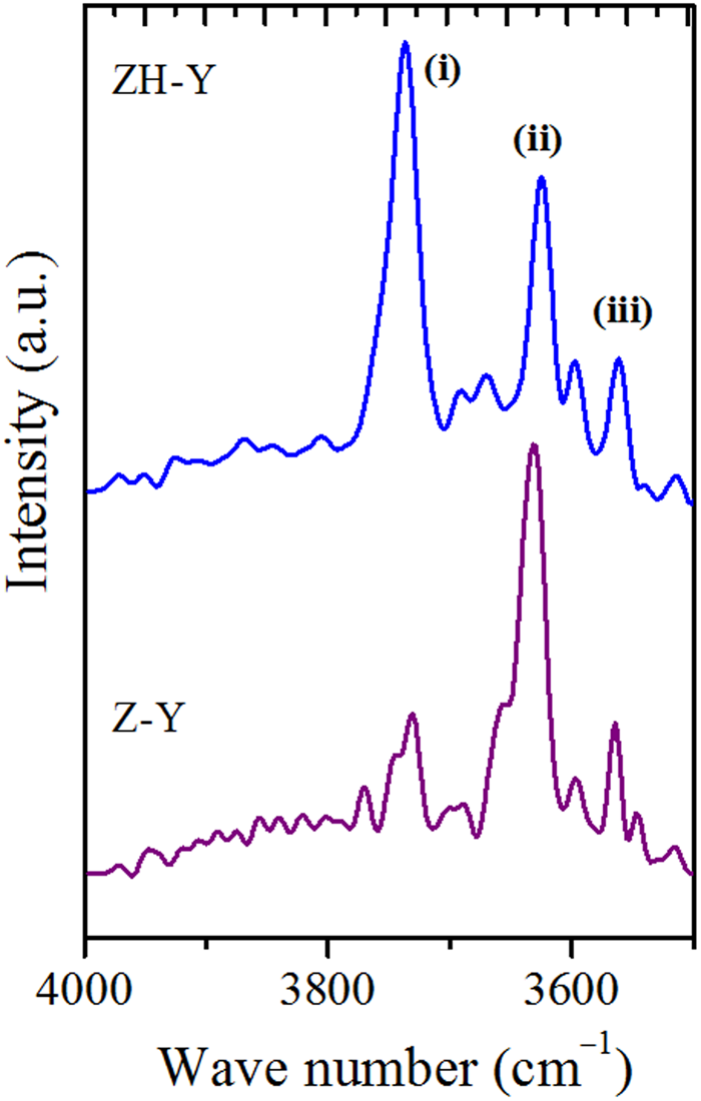
DRIFT spectra distinguishing various hydroxyl groups in zeolites.

**Figure 12:**
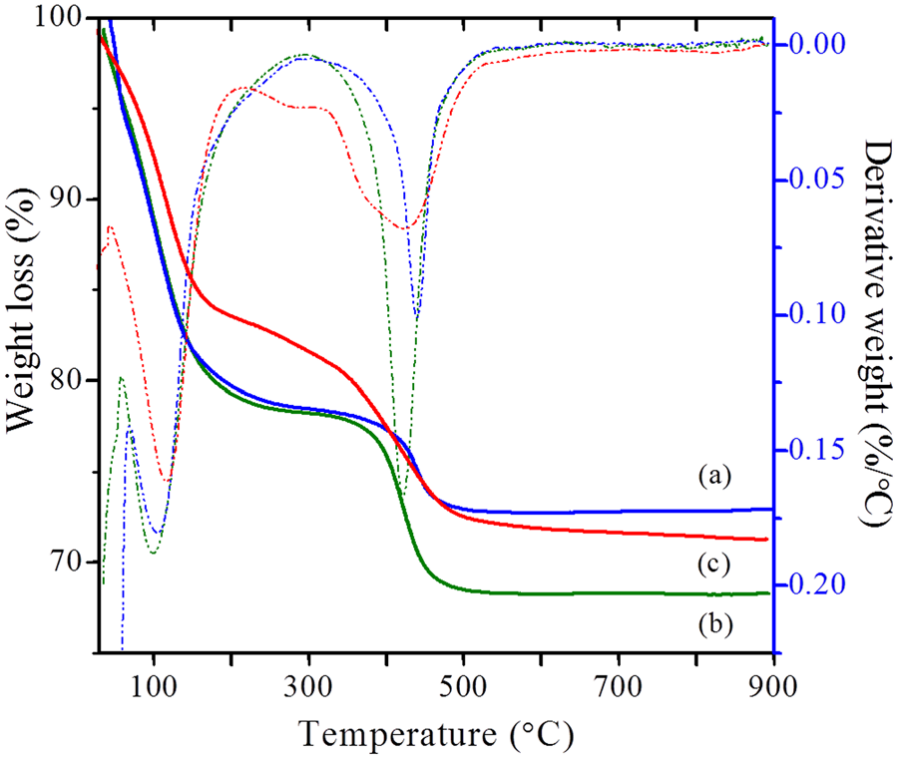
TG (solid line) and DTG (dotted line) traces of: (a) ZH-Y; (b) ZH-X; (c) ZH-A.

On the other hand, the acid properties of the synthesized zeolites have been investigated by NH_3_-TPD and pyridine DRIFTS (Fig. 10), and the obtained values are reported in Table 3. The acidity values are lesser compared to conventional counterparts. This can be attributed to the decrease in the density of acid sites due to the presence of regular mesopore channels. On the other hand, the quantitative analysis of the Brønsted (B) and Lewis acid sites and hydrogen bonded pyridine (H) was obtained by the pyridine-DRIFT spectroscopy. Furthermore, DRIFT spectra were also employed to distinguish various types of hydroxyl groups in ZH-Y and Z-Y samples (Fig. 11). The vibrations originating around 3745 *cm*^−1^ (i) can be attributed to surface silanols. Whereas, the vibrations corresponding to 3625 *cm*^−1^ (ii) and 3560 *cm*^−1^ (iii) can be due to bridged hydroxyl groups (Brønsted acid sites). On the other hand, the shoulder peaks around 3600 and 3545 *cm*^−1^ can be due to silanols arising from extra-framework species. Interestingly, the intensity of the vibrations corresponding to surface silanols at 3745 *cm*^−1^ is high in case of ZH-Y compared to Z-Y. This can be attributed to the increased surface area as well as from the silanols originating from organosilane removal which is according to the literature^47,48^. On the other hand, TG analyses (Fig. 12) of synthesized samples have revealed that the samples are thermally stable up to 900 °C. The two major weight losses around 150 °C and 450 °C can be attributed to removal of physically adsorbed water and degradation of surfactant respectively.

**Table 3:** Catalytic activities of various zeolites.

Catalyst^a^	*n*_Si_/*n*_Al_^b^	Acidity (*mmol g*^*−*1^)^c^	Phenol conversion (%)	Product selectivity (%)			TOF (*h*^*−1*^)^d^
				2-*t*-BP	4-*t*-BP	2,4-di-*t*-BP	
ZH-Y	2.2	1.76	**58.8**	4.8	**84.6**	10.6	**9.68**
Z-Y	2.3	2.26	32.4	18.7	53.5	27.8	4.15
ZH-X	1.4	1.37	**40.1**	12.5	**79.5**	7.9	**8.48**
Z-X	1.2	3.21	22.3	26.1	49.5	24.4	2.01
ZH-A	1.0	1.63	5.2	19.2	72.0	8.8	0.92
Z-A	1.0	0.87	—	—	—	—	—

^a^Reaction conditions: T = 160 °C; *t*-BA/Phenol = 2; WHSV = 7 *h*^*–1*^; TOS = 24 *h*; ^b^Determined by ^29^Si MAS-NMR; ^c^Determined by NH_3_-TPD; ^d^*n*_phenol_/(*n*_Al_.*t* (reaction time)).

### Reaction Results

**Figure 13:**
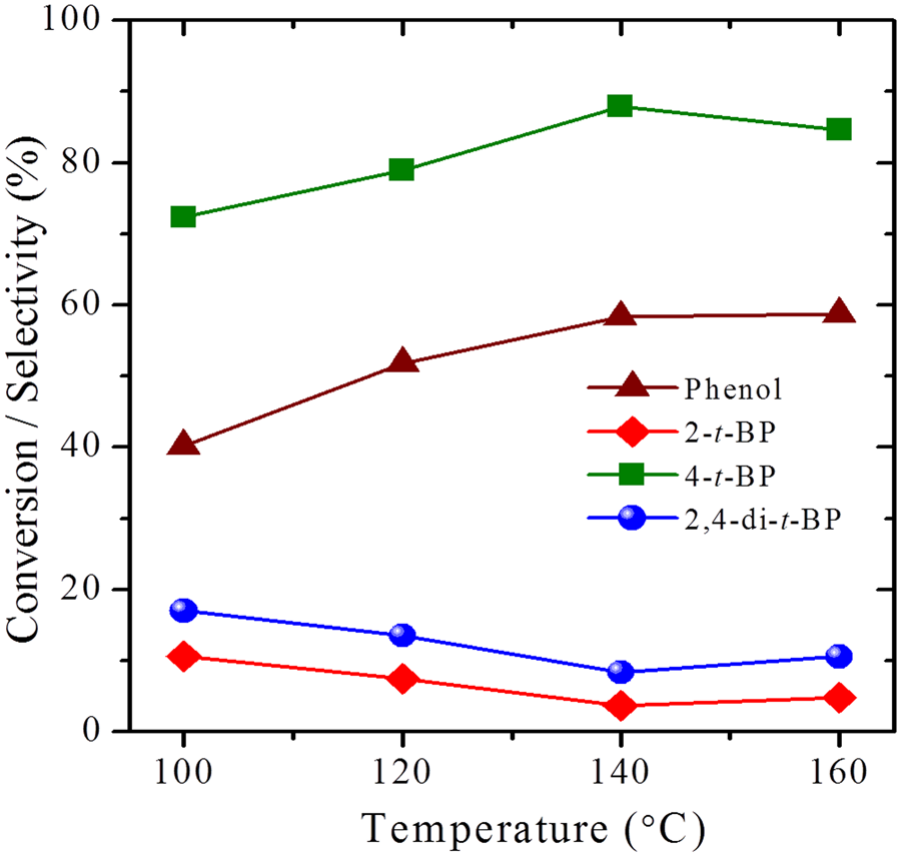
Effect of temperature on catalytic activity of ZH-Y.

**Figure 14:**
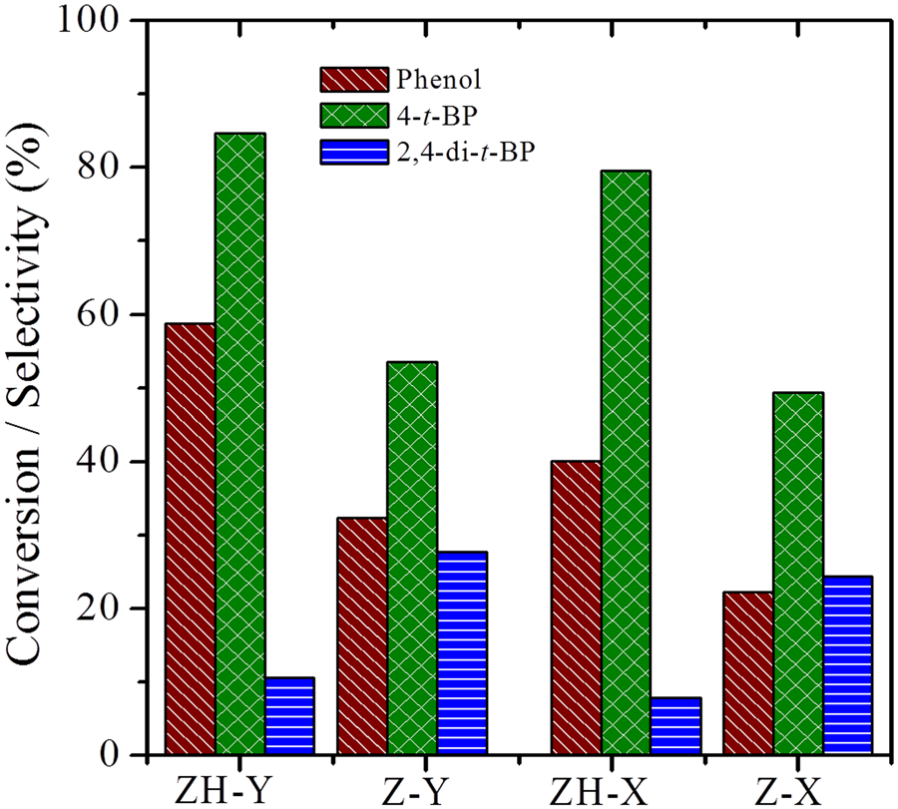
Catalytic activity studies of various zeolites for tertiary butylation of phenol.

**Figure 15:**
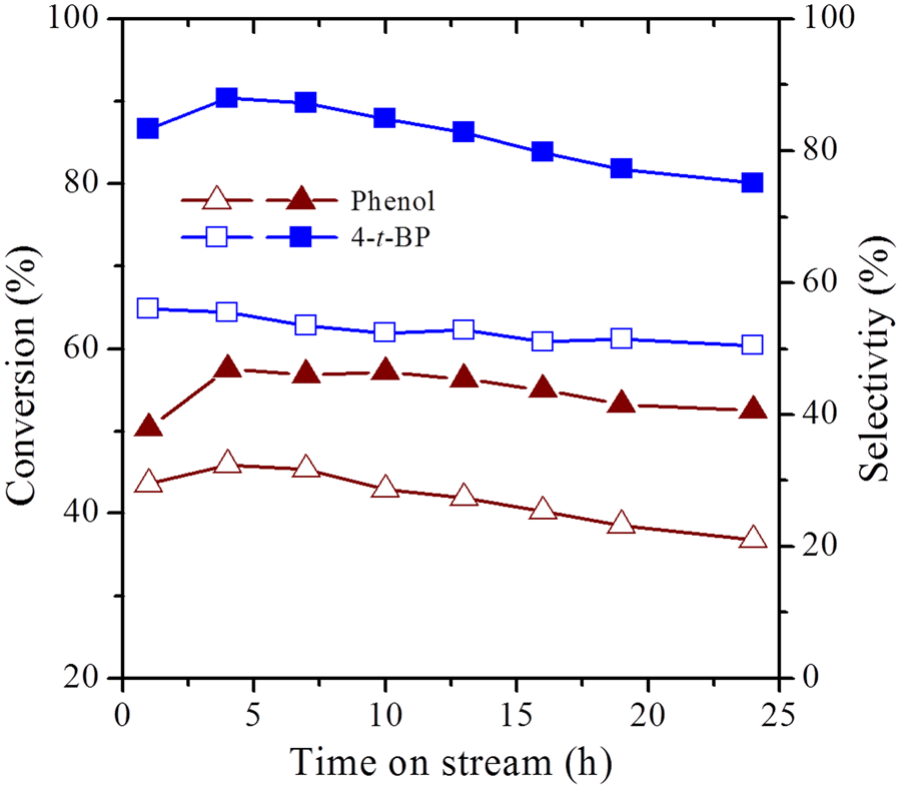
TOS studies of zeolites for tertiary butylation of phenol. Filled symbols: ZH-Y; Open symbols: Z-Y.

Consequently, the catalytic properties of the synthesized zeolites have been evaluated for industrially important vapor phase tertiary butylation of phenol (Figs 13 and 14; Table 3). In general, the selectivity of the alkylated products depends on the nature of acid sites, e.g., Brønsted acid sites preferably form 4-*t*-BP as the major product while Lewis acid sites direct the *ortho*-alkylation to form 2-*t*-BP^49–54^. Although steamed form of zeolites are the common cracking catalysts in refineries. Here, we have chosen pristine forms to improve the selectivity of 4-*t*-BP isomer, an important intermediate, which is selectively formed by acid catalysts with moderate Brønsted acidity. In this regard, the conventional zeolite, HY preferably forms 4-*t*-BP in its micropores owing to the Brønsted acidity of the catalyst. Nevertheless, the sluggish diffusion of the reactants and products in the micropore channels can lead to low conversions and distributed selectivity which are undesirable. In contrast, the hierarchical zeolites, ZH-Y and ZH-X have shown excellent conversions with a turn over frequencies much higher than that of the conventional counterparts. More importantly, the catalysts have shown outstanding selectivity (Fig. 14) of 85% and 80% towards 4-*t*-BP owing to the facile diffusion of products in the uniform mesopore channels. Furthermore, unlike the conventional zeolites, the hierarchical catalysts have shown excellent life times (Fig. 15) without much change in the efficiency up to 24 *h* of reaction stream. Further studies are in progress to test the cracking activities of the prepared zeolites in the pilot scale.

## Conclusion

In summary, the current work provides an excellent insight on the challenging, constructive synthesis of low-silica hierarchical zeolites through a stable supramolecular assembly and controlled crystallization technique. The designed pathway has led to the formation of the so-called hierarchical (nanoporous) mesostructured zeolites without scarifying the intrinsic properties of the parent zeolites. In addition, this strategy has provided a distinct self-assembly phenomenon for the formation of hierarchical LTA-type zeolites with unique radial array of mesopores. Furthermore, these hierarchical zeolites have shown superior catalytic properties with enhanced diffusion properties and excellent life-times for tertiary butylation of phenol reaction. In addition, the hierarchical zeolites can alleviate the generally encountered diffusion limitations that with the porous materials, and therefore can function as promising solid acid catalysts on industrial scale.
